# Supplementary material for: Importance of Defluviitalea raffinosedens for Hydrolytic Biomass Degradation in Co-Culture with Hungateiclostridium thermocellum
Source: Microorganisms. 2020 Jun 17;8(6):915. doi: 10.3390/microorganisms8060915 (PMC7355431; doi:10.3390/microorganisms8060915)
Supplement: Supplementary file 1 [file microorganisms-08-00915-s001.pdf]

Supplementary Materials

Table S1. Chemical analysis of the mesophilic and thermophilic inocula. Dry matter and organic dry matter were determined by drying the sample at 105 °C and incineration of the dried sample at 550 °C using standard procedures. Concentrations of volatile fatty acids (VFA, as acetic acid equivalents) were determined by steam distillation and titration with NaOH to a pH of 8.8 and the pH value according to DIN 38404-C5 and DIN 12176 standard procedures using a pH electrode. The ratio of total volatile acids/total inorganic or alkaline carbonate (TVA/TIC) was assessed as a measure of carbonate buffer capacity and process stability according to McGhee [1] using sample titration with sulfuric acid to a pH value of 5.0 and 4.4.

		inoculum	
		mesophilic	thermophilic
DM	(% FM)	9,06	13,87
oDM	(% DM)	78,93	80,41
TVA/TIC	(-)	0,23	0,37
pH	(-)	8,18	8,37
VFAs	(mg * kg_{FM}^{-1})	641,19	2025,68

DM, dry matter, oDM, organic dry matter, TVA/TIC, total volatile acids to total inorganic carbon ratio, VFAs, volatile fatty acids.

Table S2. Comparisons of the *D. raffinosedens* 16S rRNA gene sequence (MN744427) to the sequences of the related organisms deposited in the NCBI nucleotide collection (nr/nt) by means of Blastn and the Megablast algorithm.

Subject Accesion no.	Identity [%]	Alignment length [bp]	e-value	Bit score	Nucleotide source of subject [Title]
**NR_156912.1**	99.71	1396	0	2556	Anaerobic batch digester treating animal manure and rice straw [2].
**FN868420.1**	99.65	1422	0	2603	Hydrolytic bacteria for mesophilic and thermophilic biogas reactors [unpublished].
**FN868402.1**	99.65	1422	0	2601	Hydrolytic bacteria for mesophilic and thermophilic biogas reactors [unpublished].
**FN868435.1**	99.58	1422	0	2595	Hydrolytic bacteria for mesophilic and thermophilic biogas reactors [unpublished].
**HM635222.1**	99.43	1424	0	2584	Bacterial diversity of microbial community capable of lignocellulose degradation [unpublished].
**MK431708.1**	99.44	1418	0	2580	[...] enrichment and isolation of cellulolytic organisms from biogas fermenters [3].
**EU250957.1**	99.44	1418	0	2575	Composition diversity of degraded cellulose and lindane community NSC-7 [unpublished].
**EU250959.1**	99.37	1425	0	2580	Composition diversity of degraded cellulose and lindane community NSC-7 [unpublished].
**MK431707.1**	99.36	1413	0	2567	[...] enrichment and isolation of cellulolytic organisms from biogas fermenters [3].
**MK431714.1**	99.35	1380	0	2507	[...] enrichment and isolation of cellulolytic organisms from biogas fermenters [3].
**LN881573.1**	99.23	1424	0	2573	Isolation and characterization of cellulolytic bacteria from biogas plants [4].
**EU250956.1**	99.30	1422	0	2573	Composition diversity of degraded cellulose and lindane community NSC-7 [unpublished].
**EU250930.1**	99. 30	1422	0	2573	Composition diversity of degraded cellulose and lindane community NSC-7 [unpublished].
**HM635204.1**	99.24	1051	0	1897	Bacterial diversity of microbial community capable of lignocellulose degradation [unpublished].
**FN868416.1**	99.16	1423	0	2569	Hydrolytic bacteria for mesophilic and thermophilic biogas reactors [unpublished].
**EF586038.1**	99.09	1423	0	2558	Methanol-assimilating bacteria in anaerobic solid waste digester [unpublished].
**EU250932.1**	99.02	1423	0	2551	Composition diversity of degraded cellulose and lindane community NSC-7 [unpublished].
**EF558994.1**	98.88	1423	0	2543	Microbial functional groups in a thermophilic anaerobic solid waste digester revealed by stable isotope probing [unpublished].
**FN868418.1**	98.81	1424	0	2534	Hydrolytic bacteria for mesophilic and thermophilic biogas reactors [unpublished].
**FN868412.1**	98.39	1428	0	2521	Hydrolytic bacteria for mesophilic and thermophilic biogas reactors [unpublished].
**EF558948.1**	98.32	1424	0	2495	Microbial functional groups in a thermophilic anaerobic solid waste digester revealed by stable isotope probing [unpublished].

Table S3. Comparisons of the *D. raffinosedens* 16S rRNA gene sequence (MN744427) to the sequences of the related organisms deposited in the NCBI reference RNA sequence database (refseq_rna_v5) from type material only by means of Blastn and the Megablast algorithm.

Organism	Subject accession no.	Identity [%]	Alignment length	Miss-matches	e-value	bit score	Genome (y/n); Accession no.
***Defluviitalea raffinosedens* strain A6**	NR_156912.1	99.71	1396	3	0	2556	n
***Defluviitalea saccharophila* strain LIND6LT2**	NR_117912.1	96.29	1427	46	0	2338	n
***Natranaerovirga pectinivora* strain AP3**	NR_108636.1	89.91	1387	121	0	1768	y; SMAL00000000.1
***Vallitalea pronyensis* strain FatNI3**	NR_125677.1	89.81	1383	123	0	1757	n
***Abyssivirga alkaniphila* strain L81**	NR_148837.1	89.46	1385	123	0	1727	n
***Anaerostipes butyraticus* strain JCM 17466**	NR_113319.1	89.25	1395	125	0	1722	n
***Petrocella atlantisensis* strain 70B-A**	NR_164620.1	89.30	1383	129	0	1716	y; LR130778.1

Organism	Subject accession no.	Identity [%]	Alignment length	Miss-matches	e-value	bit score	Genome (y/n); Accession no.
Anaerostipes hadrus **strain DSM 3319**	NR_117139.2	89.15	1383	133	0	1707	y; AMEY00000000.1
Vallitalea guaymasensis **strain Ra1766G1**	NR_117645.1	90.18	1324	110	0	1707	y; QMDO00000000.1
Anaerocolumna cellulosilytica **strain SN021**	NR_151895.1	88.87	1392	129	0.0	1688	n
Anaerobium acetethylicum **strain GluBS11**	NR_137405.1	89.39	1357	121	0.0	1687	y; FMKA00000000.1
Herbinix luporum **strain SD1D**	NR_152095.1	88.68	1387	143	0.0	1679	y; LN879430.1
Falcatimonas natans **strain WN011**	NR_152688.1	88.61	1396	133	0.0	1674	n
Lactonifactor longoviformis **strain ED-Mt61/PYG-s6**	NR_043551.1	89.36	1344	125	0.0	1674	y; FQVI00000000.1
Parasporobacterium paucivorans **strain SYR1**	NR_025390.1	88.61	1396	132	0.0	1672	y; FQYT00000000.1
Faecalimonas umbilicata **strain EGH7**	NR_156907.1	88.66	1393	129	0.0	1670	y; SLZV00000000.1
Blautia luti **DSM 14534**	NR_114315.1	88.32	1395	141	0.0	1663	y; WMBC00000000.1
Herbinix hemicellulosilytica **strain T3/55**	NR_136763.1	88.29	1401	140	0.0	1661	y; CVTD000000000.2
Cuneatibacter caecimuris **strain BARN-424-CC-10**	NR_144608.1	88.42	1399	133	41	1422	y; SGXF00000000.1
Kineothrix alysoides **strain KNHs209**	NR_156078.1	88.62	1388	125	1	1368	y; SLUO00000000.1

Table S4. Summary of 37 complete pathway modules for annotated proteins in the genome of *D. raffinosedens* 249c-K6 as identified by means of KEGG (current status January 2020).

Pathway		Module (Module No. in KEGG)
**Carbohydrate metabolism**	Central carbohydrate metabolism	Glycolysis (Embden-Meyerhof pathway) (M00001)<br>Glycolysis (M00002)<br>Gluconeogenesis (M00003)<br>Pyruvate oxidation (M00307)<br>Citrate cycle (first carbon oxidation) (M00010)<br>Pentose phosphate pathway, non-oxidative (M00007)<br>PRPP biosynthesis (M00005)
	Other carbohydrate metabolism	Galactose degradation (Leloir pathway) (M00632)<br>Glycogen biosynthesis (M00854)<br>Nucleotide sugar biosynthesis (UDP-glucose) (M00549)<br>Nucleotide sugar biosynthesis (UDP-galactose) (M00554)
**Energy metabolism**	Carbon fixation	Phosphate acetyltransferase-acetate kinase pathway (M00579)
	Nitrogen metabolism	Nitrogen fixation (M00175)
	ATP synthesis	F-type ATPase (M00157)<br>V-type ATPase (M00159)
**Lipid metabolism**	Fatty acid metabolism	Fatty acid biosynthesis, initiation (M00082)<br>Fatty acid biosynthesis, elongation (M00083)
**Nucleotide metabolism**	Purine metabolism	Inosine monophosphate biosynthesis (M00048)<br>Adenine ribonucleotide biosynthesis (M00049)<br>Guanine ribonucleotide biosynthesis (M00050)
**Amino acid metabolism**	Serine and threonine metabolism	Serine biosynthesis (M00020)
	Cysteine and methionine metabolism	Cysteine biosynthesis (M00021)

Pathway		Module (Module No. in KEGG)
**Amino acid metabolism**		Methionine biosynthesis (M00017)
	Branched-chain amino acid metabolism	Valine/Isoleucine biosynthesis (M00019)
		Leucine biosynthesis (M00432)
	Lysine metabolism	Lysine biosynthesis (DAP dehydrogenase pathway) (M00526)
		Lysine biosynthesis (DAP aminotransferase pathway) (M00527)
	Arginine and proline metabolism<br>Arginine and proline metabolism	Ornithine biosynthesis (M00028)
		Arginine biosynthesis (M00844)
		Proline biosynthesis (M00015)
	Histidine metabolism	Histidine biosynthesis (M00026)
	Aromatic amino acid metabolism	Shikimate pathway (M00022)
		Tryptophan biosynthesis (M00023)
**Metabolism of cofactors and vitamins**	Cofactor and vitamin metabolism	NAD biosynthesis (M00115)
		Coenzyme A biosynthesis (M00120)
		C1-unit interconversion (M00140)
		Cobalamin biosynthesis (M00122)

Table S5. Summary of *D. raffinosedens* carbohydrate active enzymes including signal peptides and their possible activities as listed in the CAZy database (current status August 2019). Analysis was performed via dbCAN2 web server (National Science Foundation; http://bcb.unl.edu/dbCAN2/blast.php) [5] utilizing HMMER [6] and DIAMOND [7] including the prediction of signal peptides via SignalP 4.0 [8].

CAZyme	activities in (sub-)family	Mechanism
**GH13_36**	α-amylase and g-cyclodextrin-specific cyclodextrinase	retaining
**GH32**	invertase; endo-inulinase; β-2,6-fructan 6-levanbiohydrolase; endo-levanase; exo-inulinase; fructan β-(2,1)-fructosidase/1-exohydrolase; fructan β-(2,6)-fructosidase/6-exohydrolase; sucrose:sucrose 1-fructosyltransferase; fructan:fructan 1-fructosyltransferase; sucrose:fructan 6-fructosyltransferase; fructan:fructan 6G-fructosyltransferase; levan fructosyltransferase ; [retaining] sucrose:sucrose 6-fructosyltransferase (6-SST); cycloinulo-oligosaccharide fructanotransferase	retaining
**GH43_4**	endo-α-1,5-L-arabinanase; endo-arabinanase, arabinanase, exo-α-1,5-L-arabinanase	inverting
**GH43_16**	β-xylosidase (EC 3.2.1.37); α-L-arabinofuranosidase (EC 3.2.1.55); xylanase (EC 3.2.1.8); α-1,2-L-arabinofuranosidase (EC 3.2.1.-); exo-α-1,5-L-arabinofuranosidase (EC 3.2.1.-); [inverting] exo-α-1,5-L-arabinanase (EC 3.2.1.-); β-1,3-xylosidase (EC 3.2.1.-); [inverting] exo-α-1,5-L-arabinanase (EC 3.2.1.-); [inverting] endo-α-1,5-L-arabinanase (EC 3.2.1.99); exo-β-1,3-galactanase (EC 3.2.1.145); β-D-galactofuranosidase (EC 3.2.1.146)	inverting
**GH43_22**	β-xylosidase ; arabinofuranosidase	inverting
**CBM6**	Modules of approx. 120 residues. The cellulose-binding function has been demonstrated in one case on amorphous cellulose and β-1,4-xylan. Some of these modules also bind β-1,3-glucan, β-1,3-1,4-glucan, and β-1,4-glucan.	-
**CBM38**	The inulin-binding function has been demonstrated in the case of the cycloinulo-oligosaccharide fructanotransferase from *Paenibacillus macerans (Bacillus macerans*) by Lee et al. [9].	-

Table S6. α-Diversity of biogas-producing communities originating from two mesophilic and two thermophilic biogas reactors under different process conditions as deduced from 16S rDNA and reverse transcribed 16S rRNA amplicon sequences. Next generation sequencing of the bacterial 16S rDNA and reverse transcribed 16S rRNA, hypervariable gene regions V6-V8, was performed with Illumina MiSeq at Core-Facility Microbiome/NGS, ZIEL—Institute for Food & Health, TU Munich, Freising, Germany. Analysis of the sequencing data was performed at CeBiTec, Bielefeld, Germany. The statistical calculation was performed via RHEA [10] in R.

Fermenter	Temperature	Process condition	Nucleic acid	Richness	Shannon	Shannon.effective	Simpson	Simpson.effective	Evenness
**1**	38	stable	DNA	371	3.56	35.22	0.07	13.87	0.42
**1**	38	stable	cDNA	391	4.01	55.16	0.04	27.59	0.47
**1**	38	acidified	DNA	298	3.09	21.93	0.10	10.43	0.38
**1**	38	acidified	cDNA	269	3.35	28.62	0.10	9.79	0.42
**2**	38	stable	DNA	381	3.53	34.04	0.08	12.90	0.41
**2**	38	stable	cDNA	354	4.02	55.71	0.04	28.21	0.47
**2**	38	acidified	DNA	260	2.98	19.69	0.13	7.60	0.37
**2**	38	acidified	cDNA	311	3.57	35.39	0.08	13.28	0.43
**1**	50	stable	DNA	274	2.72	15.17	0.22	4.60	0.34
**1**	50	stable	cDNA	189	2.69	14.67	0.14	7.31	0.36
**1**	50	highly efficient	DNA	244	2.88	17.82	0.14	7.09	0.36
**1**	50	highly efficient	cDNA	209	2.68	14.53	0.14	7.32	0.35
**2**	50	stable	DNA	289	2.83	16.95	0.19	5.32	0.35
**2**	50	stable	cDNA	219	2.75	15.60	0.14	7.16	0.35
**2**	50	highly efficient	DNA	271	2.96	19.25	0.13	7.93	0.37
**2**	50	highly efficient	cDNA	200	2.72	15.21	0.13	7.56	0.36
			**Average**	283	3.15	25.94	0.12	11.12	0.39

Table S7. Relative abundance of the genus *Defluviitalea* in two mesophilic and two thermophilic biogas reactor sludge at different process conditions. Next generation sequencing of the bacterial 16S rDNA and reverse transcribed 16S rRNA, hypervariable gene regions V6-V8, was performed with Illumina MiSeq at Core-Facility Microbiome/NGS, ZIEL—Institute for Food & Health, TU Munich, Freising, Germany. Analysis of the sequencing data was performed at CeBiTec, Bielefeld, Germany. The statistical calculation was performed via RHEA [10] in R. Results are shown in ascending order for the relative abundance of *Defluviitalea* [%].

Replicate	Process condition	Nucleic acid	Relative abundance of *Defluviitalea* [%]
**1**	m_ac	cDNA	0.00
**2**	m_st	DNA	0.00
**1**	m_st	cDNA	0.00
**1**	m_st	DNA	0.01
**2**	m_st	cDNA	0.01
**1**	m_ac	DNA	0.02
**2**	m_ac	cDNA	0.07
**2**	m_ac	DNA	0.10
**2**	t_st	cDNA	0.22
**1**	t_st	DNA	0.26
**2**	t_st	DNA	0.26
**1**	t_ef	DNA	0.31
**1**	t_ef	cDNA	0.45
**2**	t_ef	DNA	0.72
**1**	t_st	cDNA	0.81
**2**	t_ef	cDNA	1.44

m_st, mesophilic, stable; m_ac, mesophilic, acidified; t_st, thermophilic (50 °C), stable; t_ef, thermophilic, highly efficient.

Table S8. Abundance of *D. raffinosedens* in two mesophilic and two thermophilic biogas reactor sludge at different process conditions. Next generation sequencing of the bacterial 16S rDNA and reverse transcribed 16S rRNA, hypervariable gene regions V6-V8, was performed with Illumina MiSeq at Core-Facility Microbiome/NGS, ZIEL—Institute for Food & Health, TU Munich, Freising, Germany. Analysis of the sequencing data as well as the mapping to the references *D. raffinosedens*, 249c-K6 was performed at CeBiTec, Bielefeld, Germany.

Replicate	Process condition	Nucleic acid	Number of hits	reads in total	percentage [%]
**1**	m_st	DNA	0	36,061	-
**2**	m_st	DNA	0	42,433	-
**1**	m_ac	DNA	0	39,518	-
**2**	m_ac	DNA	0	40,029	-
**1**	t_st	DNA	8	36,076	0.02
**2**	t_st	DNA	0	36,211	-
**1**	t_ef	DNA	36	42,417	0.08
**2**	t_ef	DNA	103	31,044	0.33
**1**	m_st	cDNA	0	27,054	-
**2**	m_st	cDNA	0	18,571	-
**1**	m_ac	cDNA	0	17,775	-
**2**	m_ac	cDNA	0	34,050	-
**1**	t_st	cDNA	64	24,340	0.26
**2**	t_st	cDNA	0	33,053	-
**1**	t_ef	cDNA	54	31,362	0.17
**2**	t_ef	cDNA	203	29,471	0.69
		**Sum**	**468**	**519465**	

Hits, minimum mapping length of 300 bases and a minimum percent identity of 99% to the full-length 16S rRNA gene sequence of *D. raffinosedens* 249c-K6 accession no MT350287.

m_st, mesophilic, stable; m_ac, mesophilic, acidified; t_st, thermophilic, stable; t_ef, thermophilic, highly efficient.

References Supplementary Materials: .

1. McGhee, J. A method for approximation of volatile acid concentrations in anaerobic digesters. Water and Sewage Works **1968**, *115*, 162–166.
2. Ma, S.; Huang, Y.; Wang, C.; Fan, H.; Dai, L.; Zhou, Z.; Liu, X.; Deng, Y. *Defluviitalea raffinosedens* sp. nov., a thermophilic, anaerobic, saccharolytic bacterium isolated from an anaerobic batch digester treating animal manure and rice straw. *Int J Syst Evol Microbiol* **2017**, *67*, 1607.
3. Rettenmaier, R.; Duerr, C.; Neuhaus, K.; Liebl, W.; Zverlov, V.V. Comparison of sampling techniques and different media for the enrichment and isolation of cellulolytic organisms from biogas fermenters. *Syst Appl Microbiol* **2019**.
4. Koeck, D. Isolierung und Charakterisierung von cellulolytischen Bakterien aus der mikrobiellen Lebensgemeinschaft in Biogasanlagen. Technische Universität München, 2015.
5. Zhang, H.; Yohe, T.; Huang, L.; Entwistle, S.; Wu, P.; Yang, Z.; Busk, P.K.; Xu, Y.; Yin, Y. dbCAN2: A meta server for automated carbohydrate-active enzyme annotation. *Nucleic Acids Res* **2018**, *46*, W95¨CW101.
6. Finn, R.D.; Clements, J.; Eddy, S.R. HMMER web server: Interactive sequence similarity searching. *Nucleic Acids Res* **2011**, *39*, W29¨CW37.
7. Buchfink, B.; Xie, C.; Huson, D.H. Fast and sensitive protein alignment using DIAMOND. *Nat. Methods* **2015**, *12*, 59.
8. Petersen, T.N.; Brunak, S.; Von Heijne, G.; Nielsen, H. SignalP 4.0: Discriminating signal peptides from transmembrane regions. *Nat. Methods* **2011**, *8*, 785.
9. Lee, J.-H.; Kim, K.-N.; Choi, Y.-J. Identification and characterization of a novel inulin binding module (IBM) from the CFTase of Bacillus macerans CFC1. *FEMS Microbiol. Lett.* **2004**, *234*, 105–110.
10. Lagkouvardos, I.; Fischer, S.; Kumar, N.; Clavel, T. Rhea: A transparent and modular R pipeline for microbial profiling based on 16S rRNA gene amplicons. PeerJ 5: e2836. 2017.
